# Associations between relative abundances of *Bifidobacterium* species in the gut and DNA methylation of cortisol-related genes in a pediatric population

**DOI:** 10.3389/fmicb.2025.1558809

**Published:** 2025-09-22

**Authors:** Samantha A. Harker, Kevin S. Bonham, Shelley Hoeft McCann, Alexandra R. Volpe, Qiyun Zhu, Viren D’Sa, Daphne Koinis-Mitchell, Sean C. L. Deoni, Vanja Klepac-Ceraj, Candace R. Lewis

**Affiliations:** ^1^School of Life Sciences, Arizona State University, Tempe, AZ, United States; ^2^Department of Biological Sciences, Wellesley College, Wellesley, MA, United States; ^3^Department of Pediatrics, Warren Alpert Medical School at Brown University, Providence, RI, United States; ^4^Bill & Melinda Gates Foundation, Seattle, WA, United States; ^5^Neurogenomics Division, Translational Genomics Research Institute (TGen), Phoenix, AZ, United States

**Keywords:** gut microbiome, *Bifidobacterium*, *Bifidobacteria*, DNA methylation, epigenetics, folate, HPA

## Abstract

**Introduction:**

Human epigenetics, specifically DNA methylation, and the gut microbiome are dynamic systems influenced by environmental factors, such as diet and earlylife exposures, with profound implications for health and disease. Metabolites produced by the gut microbiome interact with the host, shaping physiological processes. While prior research has linked *Bifidobacterium* abundance to anxiety and cortisol function, the role of DNA methylation as a potential mechanism underlying these associations remains unexplored. This study examines the relationship between the relative abundance of *Bifidobacterium* species in the gut and DNA methylation of hypothalamic–pituitary–adrenal (HPA) axis genes in a pediatric cohort. We hypothesized that *Bifidobacterium* abundance would predict DNA methylation at key HPA genes associated with stress response, including *NR3C1*, *FKBP5*, and more.

**Methods:**

Multiple linear regression and regularized canonical correlation analysis (rCCA) were used.

**Results:**

There were significant associations between *Bifidobacterium* abundance and DNA methylation at HPA gene loci, while control analyses showed no association with global methylation levels. rCCA further pinpointed specific *Bifidobacterium* species, such as *B. angulatum* and *B. adolescentis*, as strong contributors to the first canonical component, correlating with CpG sites influencing HPA gene methylation.

**Discussion:**

These findings suggest that microbiome-derived metabolites, such as folate, may modulate DNA methylation and stress physiology. This work provides new insights for exploring how the gut microbiome impacts mental health and stress resilience, offering potential pathways for microbiome-targeted interventions.

## 1 Introduction

Epigenetics, encompassing the biomolecular processes that regulate gene expression without altering the underlying DNA sequence, has emerged as a key player in health and disease. This dynamic system has been linked to a wide array of conditions, including cancer (Dawson and Kouzarides, 2012), obesity and diabetes (Ling and Rönn, 2019), as well as developmental (Zhao et al., 2007), psychiatric (Peña et al., 2014), and neurodegenerative disorders (Migliore and Coppedè, 2009). Critically, certain life stages, such as embryonic development (Kessler et al., 2018), early childhood (Lewis and Olive, 2014), and adolescence, appear to be particularly susceptible windows during which epigenetic programming is shaped by environmental exposures such as diet, environmental toxins, maternal behavior, or childhood abuse (Alegría-Torres et al., 2011; Niwa et al., 2016; Ochi and Dwivedi, 2023). Similarly, the microbiome, the community of microorganisms residing within and on the human body, also plays an important role in host physiology and health (Shreiner et al., 2015). Gut microbiome composition also has associations with cancer (Schwabe and Jobin, 2013), metabolic diseases such as obesity and diabetes (Devaraj et al., 2013), and a range of developmental (Vuong and Hsiao, 2017), psychiatric (Kelly et al., 2015), and neurodegenerative disorders (Roy Sarkar and Banerjee, 2019). Notably, many of the same early life exposures that influence the epigenetic programming also impact gut microbiome composition and function, highlighting an intricate interplay between the two systems (Dong and Gupta, 2019).

Early-life stress (ELS) impacts hypothalamic–pituitary–adrenal (HPA) axis function, at least partially, through epigenetic regulation, particularly DNA methylation (Parade et al., 2021). In parallel, substantial evidence from animal studies demonstrates that ELS alters gut microbiome composition, which subsequently influences HPA function, contributing to anxiety and depression-like behaviors (Bailey et al., 2004; Rea et al., 2016; Dinan and Cryan, 2012; Hantsoo and Zemel, 2021). Emerging research suggests similar effects in humans (Rosin et al., 2021; Keskitalo et al., 2021; Vogel et al., 2020; Flannery et al., 2020). Although bidirectional communication between the brain and gut microbiome—via neural, endocrine, and inflammatory pathways—is well established, the specific mechanisms remain unclear (Osadchiy et al., 2019). A compelling hypothesis is that ELS-induced changes in the gut microbiome alter levels of biosynthesized metabolites, which in turn regulate host epigenetic processes affecting HPA function (Kok et al., 2018; Louwies et al., 2020; Marín-Tello et al., 2022).

Folate, also known as vitamin B9, plays an essential role in cellular function and is a key regulator of epigenetic processes. It drives one-carbon metabolism, a sophisticated network of metabolic pathways that provide the methyl groups necessary for DNA methylation (McKay and Mathers, 2011). Folate deficiency can lead to aberrant DNA methylation and disease etiology (Crider et al., 2012). Folate cannot be synthesized by mammals and must, therefore, be obtained from other sources. Interestingly, 13% of microbiome reference genomes contain all genes required for folate synthesis. An additional 39% of microbiome genomes can synthesize folates in the presence of pABA, an upstream intermediate obtained through diet or from other intestinal microbes (Engevik et al., 2019). Dietary folate is primarily absorbed in the small intestine, while folate produced by gut bacteria enters the bloodstream via transporters in the colon (Magnúsdóttir et al., 2015; Said, 2011; Yoshii et al., 2019). Animal studies estimate that at least 18% of circulating folate originates from bacterial production (Asrar and O’Connor, 2005; Park et al., 2013). The presence of colon folate transporters and the higher absorption rate of colon-derived folate compared to dietary intake suggest a significant role of bacterially synthesized folate in host physiology (Qiu et al., 2006; Lakoff et al., 2014). Consequently, the composition of the host microbiome and the abundance of folate-producing bacterial strains may influence bioavailable folate and impact epigenetic processes, such as DNA methylation.

Multiple studies suggest that microbiome-mediated effects on the host epigenome play a role in various disease states, including cancer (Asrar and O’Connor, 2005), immune-mediated disorders (Zheng et al., 2020), inflammatory bowel disease (Aleksandrova et al., 2017), and obesity and diabetes (Sharma et al., 2020). This study builds on previous research by examining the association between the genus *Bifidobacterium* and HPA gene DNA methylation in a healthy pediatric cohort. *Bifidobacteria* are among the most beneficial gut microbiota, contributing significantly to host health through functions such as folate production (Rossi et al., 2011). Numerous studies in rodent models have demonstrated the anxiolytic, antidepressant, and HPA-axis-modulating effects of *Bifidobacteria* (Yunes et al., 2020), with an expanding body of evidence supporting similar findings in humans (Altaib et al., 2021; Akkasheh et al., 2016).

In this study, we hypothesized that the relative abundance of *Bifidobacterium* species would predict DNA methylation patterns in key HPA-axis genes previously linked to early life stress, including *NR3C1*, *FKBP5*, *AVP*, *CRH*, *CRHR1*, and *CRHR2*. To test these hypotheses, we employed two analytical approaches: a traditional multiple linear regression model and regularized canonical correlation analysis (rCCA) (Leurgans et al., 1993). rCCA is an integrative, correlation-based method that identifies latent features shared across multimodal datasets. This approach facilitates a systems biology perspective, bypasses the need for multiple hypothesis testing, and accounts for numerous small effect sizes. rCCA is particularly well-suited for scenarios where the number of measured features exceeds the sample size, as is typical in modern omics studies (González et al., 2009). Understanding these associations during early life is especially critical, as both the microbiome and the epigenome are highly responsive to environmental influences during this developmental stage.

## 2 Methods

### 2.1 Parent study

Our study was based on a subset of participants prospectively followed as part of the Environmental influences on Child Health Outcomes (ECHO) Program. ECHO is a consortium of 69 established pediatric cohort studies collecting new data under a common protocol since 2019 (Gillman and Blaisdell, 2018) with the primary aim to study the effects of early-life environmental exposures on child health. Single and cohort-specific institutional review boards monitored human subject activities and the centralized ECHO Data Analysis Center. All participants provided written informed consent.

Eligibility criteria for the parent study included mothers >18 years old, term gestation: 37–41 weeks, healthy singleton pregnancy, no evidence of uncontrolled medical conditions (i.e., hypertension, pre-eclampsia, uncontrolled diabetes) or medical conditions that could potentially impact the safety of a participant during a study visit, no history of major psychiatric illness, English speaking, consent to baby brain imaging, and the longitudinal nature of the study; infants had no significant congenital anomalies, and infants had no history of neurological trauma or disorder (e.g., epilepsy). Inclusion criteria for the subset used in this study included participants who came to the lab during the study recruitment period. Participants’ recruitment occurred in person at a research visit or remotely.

### 2.2 Demographics

Demographic information was collected by parental report and is summarized in Table 1 and Supplementary Figures 1, 2. Our sample size was determined by the largest number of eligible participants available to us within the ECHO dataset, given the study’s inclusion/exclusion criteria. This approach maximized statistical power while ensuring that all available relevant data were utilized. To reduce temporal variability across biospecimen types while maintaining a well powered sample from an extant dataset, only participants with fecal and saliva samples collected within 365 days of one another were included in the analyses (M = 77.5 days; SD = 110). To account for missing data on age (*n* = 30 out of *N* = 142), we employed data imputation with the R package “mice” using the pmm method, which produced the most similar age distribution and did not change the overall age range. After imputation, age ranged from 1 month to 15 years old (*N* = 142; *M*_age_ = 4.27, SD = 3.89), and 40% were females. No other variables were imputed. 76% of the participants reported non-Latino/Hispanic ethnicity, and 77.5% of the participants reported White as their race. Race and ethnicity were determined via parent report. Written consent was obtained from parents or legal guardians in accordance with ethics approval from the host institution’s Institutional Review Board.

**Table 1:** Participant demographics.

Age		
	Mean ± SD	4.27 ± 3.89
	Range	1 m–15y
Sex (%)		
	Male	59.2
	Female	40.8
Ethnicity (%)		
	Hispanic/Latino	23.9
	Non-Hispanic/Latino	76.1
Race (%)		
	Asian	0.70
	Asian Indian	1.41
	Black or African American	5.63
	Black or African American\American Indian or Alaska Native	0.70
	White	77.50
	White\Black or African American	3.52
	White\Black or African American\American Indian or Alaska Native	0.70
	White\Other Asian	0.70
	Mixed race	3.52
	Unknown	4.23
	Decline to answer	1.41

Summary of participant demographic information.

### 2.3 Stool and saliva collection

Saliva was collected from participants in the lab using Oragene (DNA Genotek, Ottawa, Ontario, Canada) saliva collection kits. DNA was extracted with a standard isolation kit (DNA Genotek’s PT-L2P-5). Sample yield and purity were assessed spectrophotometrically using NanoDrop ND-1000 (ThermoScientific, Wilmington, DE) methods. Stool samples were collected by parents in OMR-200 tubes (OMNIgene GUT, DNA Genotek, Ottawa, Ontario, Canada), stored on ice, and brought within 24 h to the laboratory in RI, where they were immediately frozen at −80°C. Stool samples were not collected if the infant had taken antibiotics within the last two weeks. Samples were transported to Wellesley College (Wellesley, MA) on dry ice for further processing. Nucleic acids were extracted from 200 μL of each stool sample using the RNeasy PowerMicrobiome kit automated on the QIAcube (Qiagen, Germantown, MD), excluding the DNA degradation steps. Cell lysing steps were performed using the Qiagen PowerLyzer 24 Homogenizer (Qiagen, Germantown, MD) at 2500 speed for 45 s, then samples were transferred to the QIAcube to complete the protocol, and extracted DNA was eluted in a final volume of 100 μL.

### 2.4 Sequencing of metagenomes

Extracted DNA was sequenced at the Integrated Microbiome Resource (IMR, Dalhousie University, NS, Canada) (Aleksandrova et al., 2017). To sequence metagenomes, a pooled library (max 96 samples per run) was prepared using the Illumina Nextera Flex Kit for MiSeq and NextSeq (a PCR-based library preparation procedure) from 1 ng of each sample, where samples were enzymatically sheared and tagged with adaptors, PCR amplified while adding barcodes, purified using columns or beads, and normalized either using Illumina beads or manually. Samples were then pooled onto a plate and sequenced on the Illumina NextSeq 550 platform using 150 + 150 bp paired-end “high output” chemistry, generating ~400 million raw reads and ~120 Gb of sequence. Samples were deposited in NCBI GenBank under BioProject PRJNA695570. Mean read depth across all samples in this study was 7,841,324, with a standard deviation of 3,409,844.

### 2.5 DNA methylation microarrays

DNA was treated with sodium bisulfite using the EZ-96 DNA Methylation Kit (Zymo Research, Irvine, CA). DNA methylation was quantified using the Infinium MethylationEPIC BeadChip run on an Illumina iScan System (Illumina, San Diego, CA). Raw IDAT files were exported for preprocessing in R with the minfi package, and standard quality control analyses were performed, including quantile normalization, checking for sex mismatches, and excluding low-intensity samples (*p* detection < 0.01) (Aryee et al., 2014). Three samples did not pass our quality control pipeline due to low intensity. Using the R package EpiDISH (Epigenetic Dissection of Intra-Sample Heterogeneity, 3.8) RPC method, we estimated the proportion of epithelial cells per sample.

### 2.6 Analyzing metagenomes

Metagenomic data were analyzed using the bioBakery workflow with all necessary dependencies and default parameters (McIver et al., 2018). Briefly, KneadData (v0.7.10) was used to trim and filter raw sequence reads and to separate human and 16S ribosomal rRNA gene reads from bacterial sequences in both fecal and oral samples. Samples that passed quality control were taxonomically profiled to the genus level using MetaPhlAn (v3.0.7) (Beghini et al., 2021).

### 2.7 *Bifidobacterium* composite

We created a composite variable by summing across all *Bifidobacterium* strains measured in our sample (Supplementary Table 1). Relative abundance values were normalized within individual participants such that the total sum equaled 100%. For each composite, we summed the normalized relative abundances that belonged to the same genus-level group, *Bifidobacterium*. This summation approach was used to reduce dimensionality and to reflect total genus-level abundance, allowing us to focus on broader microbial patterns rather than individual taxa that may be highly sparse.

### 2.8 Statistical analyses

#### 2.8.1 Principal component analyses

A commonly used method to detect patterns in DNA methylation data is principal component analysis (PCA) (Sharma et al., 2020; Rossi et al., 2011; Yunes et al., 2020; Altaib et al., 2021), a dimensionality reduction procedure (Akkasheh et al., 2016). PCA is used to develop a smaller number of latent variables, called principal components, with the first principal component (PC1) accounting for the most variance in the observed variables (Akkasheh et al., 2016). To address the multiple testing burden associated with analyzing numerous CpG sites per gene of interest and to reduce the risk of Type II error, we applied principal component analysis (PCA) to all CpG sites annotated to each gene of interest. PCA was conducted within each gene separately, using methylation values from individual CpG sites annotated to that gene. This approach allowed us to capture the major axes of variability in methylation within each gene while reducing dimensionality and multicollinearity among sites. We acknowledge that this method may reduce site-specific interpretability but prioritized gene-level summaries to facilitate downstream analyses and reduce Type I error. The first and second principal components (PC1; PC2) of each gene were used as outcome variables in regression models (Supplementary Table 2).

#### 2.8.2 Multiple linear regression

The *Bifidobacterium* composite was used as a predictor variable in multiple linear regression models while controlling for sex, age, sequencing depth, and time between saliva and fecal sample collection. Importantly, we did not include the estimated epithelial cell count percentage as a covariate due to high multicollinearity with all PC1s, which has been found by other groups (Leurgans et al., 1993). We also created a proxy measure of global methylation by first defining the top 50% variable CpG sites across samples and averaging them together per individual (the variable is referred to as ‘Global50p’). This variable was used as a control analysis to assess if *Bifidobacterium* levels are associated with DNA methylation globally or specifically to our genes of interest. We limited global DNA methylation analysis to the top 50% most variable CpG sites to reduce dimensionality, improve interpretability, and focus on sites with greater biological variability, which are more likely to reflect meaningful differences across individuals. This approach is commonly used in epigenomic studies to enrich for signal over noise and to increase statistical power in downstream analyses. To more closely evaluate the relationships between *Bifidobacterium* and DNA methylation, we assessed the location of CpG sites with the largest loading values onto the first principal component.

#### 2.8.3 Association-based mediation analyses

Given that both the microbiome and epigenome exhibit significant variability during early development, age could act as a confounding variable influencing associations between the two. To address this, we incorporated age as a covariate in all models. Furthermore, to explicitly evaluate the role of age, we performed an association-based mediation analysis to quantify the proportion of the relationship between *Bifidobacterium* abundance and HPA gene DNA methylation that is mediated by age.

#### 2.8.4 Regularized canonical correlation analysis (rCCA)

Regularized canonical correlation analysis (rCCA) was used to identify a set of orthogonal linear combinations, or canonical (latent) variates, that maximized the shared variability between two datasets. Components (canonical variates) for each dataset were calculated separately and optimized to maximize the correlation between the corresponding variates. rCCA was selected as an alternative to multiple regression given the presence of multiple intercorrelated outcome variables.

A scree plot (Supplementary Figure 3) was generated, and the elbow point was identified at dimension = 3, where the decrease in canonical correlation notably slowed. Accordingly, we also tested ncomp = 3 to assess whether results differed substantially. To further validate findings, cross-validation was performed using the tune.rcc function in the mixOmics R package to optimize the Ridge regularization parameters (lambda1, lambda2). Default settings were applied, which implemented 10-fold leave-one-out cross-validation. The optimal parameters returned were lambda1 = 1 and lambda2 = 0.7503, which were then used in the rcc function with method = “ridge” (Supplementary Figure 4). These results were compared to those obtained using the shrinkage method.

The analysis included 142 samples and two datasets: X (*Bifidobacterium* abundance, 9 variables) and Y (*NR3C1* CpG site DNA methylation, 113 variables). Both datasets were preprocessed using the shrinkage method to ensure optimal regularization. Strains with a total sum count < 1 across all individuals were excluded (Supplementary Table 1). The regularization parameters applied were 0.7637 for the X dataset and 0.0564 for the Y dataset. The rcc function (mixOmics) was then used to perform rCCA with a correlation threshold of 0.3, extracting two components for further analysis.

Statistical significance was evaluated via permutation testing with 10,000 iterations, randomly shuffling sample labels of one dataset to generate a null distribution of canonical correlations. To aid interpretation of canonical variates, loadings were examined to identify the most influential CpGs and strains contributing to observed associations, focusing on features with absolute correlation coefficients > 0.3. This threshold was determined to balance interpretability with statistical rigor in detecting moderate associations.

## 3 Results

### 3.1 Relative abundance of *Bifidobacterium* significantly predicts HPA-gene DNA methylation

**Figure 1:**
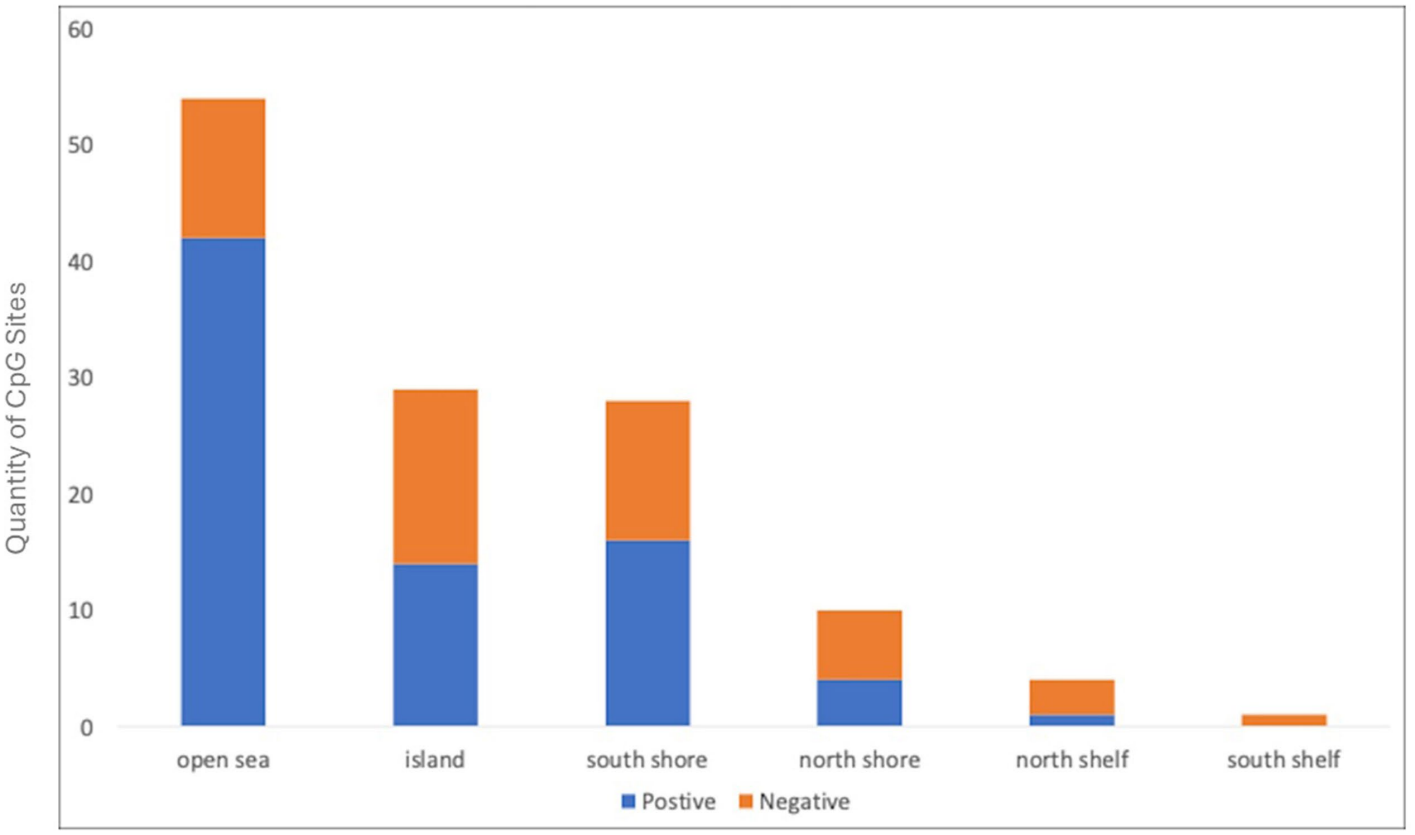
Directionality and CpG site location of HPA-gene DNA methylation and *Bifidobacterium* abundance. Graph depicts the number of CpG sites and their gene location with a significant positive (blue) or negative (orange) relationship with *Bifidobacterium* relative abundance.

The *Bifidobacterium* composite variable was a significant predictor of DNA methylation Principal Component 1 (PC1) of all genes except *CRH*. In contrast, the *Bifidobacterium* composite only predicted one PC2, specifically *CRH* PC2 (Table 2; Supplementary Figure 5). In the control analysis, *Bifidobacterium* was not a significant predictor of global50p DNA methylation (b = −8.231e-04, *p* = 0.11). Principal component analysis (PCA) calculates correlations between CpG sites and the first principal component (PC1), referred to as loading values. CpG sites correlated with PC1 exhibit co-variation; when one site increases in methylation, the others increase or decrease proportionally. Consequently, PC1s represent higher methylation values at CpG sites with positive loadings and lower methylation at sites with negative loadings. In our analysis, the PC1s were mostly influenced by positive loadings (Figure 1).

**Table 2:** *Bifidobacterium* significantly predicts HPA gene DNA methylation omnibus and PCA results for HPA genes predicting *Bifidobacterium.*

	Omnibus results			PC1		PC2	
Gene	*F*	*p*	R2	B	*p*	B	*p*
*NR3C1*	52.16	<2.2e-16	0.60	−0.17	0.002	−0.02	0.76
*FKBP5*	46.41	<2.2e-16	0.58	0.19	0.00	−0.08	0.24
*AVP*	42.98	<2.2e-16	0.56	0.19	0.00	−0.11	0.13
*CRH*	6.59	7.06e-05	0.16	−0.14	0.08	0.23	0.00
*CRHR1*	53.01	<2.2e-16	0.61	−0.18	0.00	0.06	0.45
*CRHR2*	42.72	<2.2e-16	0.56	−0.15	0.01	−0.02	0.77

Multiple R2 is reported. DF for every model = 4, 137.

### 3.2 Age does not fully account for the associations between *Bifidobacterium* and HPA-gene DNA methylation

The relationship between PC1 DNA methylation of all genes (and *CRH* PC2) with *Bifidobacterium* was significantly mediated by age; the proportion mediated ranged from 57–62% (Table 3; full mediation results are included in Supplementary Table 3). These results demonstrate that while age is a significant factor driving the association between *Bifidobacterium* and DNA methylation, it does not fully mediate the relationship. It is known that *Bifidobacterium* presence reduces with age; thus, we also explored the interaction of age and *Bifidobacterium* abundance. We did not find any significantly meaningful results.

**Table 3:** HPA gene DNA methylation and *Bifidobacterium* proportion mediated by age.

*Bifidobacterium*				
Gene	Estimate	95% CI lower	95% CI upper	*p*-value
*NR3C1* (PC1)	0.60	0.46	0.77	<2e-16
*FKBP5* (PC1)	0.57	0.42	0.74	<2e-16
*AVP* (PC1)	0.58	0.43	0.77	<2e-16
*CRH* (PC2)	0.50	0.33	0.74	<2e-16
*CRHR1* (PC1)	0.57	0.44	0.72	<2e-16
*CRHR2* (PC1)	0.62	0.46	0.80	<2e-16

Mediation results for HPA gene DNA methylation and *Bifidobacterium* mediated by age.

### 3.3 There are strong canonical correlations between *Bifidobacterium* and *NR3C1* DNA methylation

**Figure 2:**
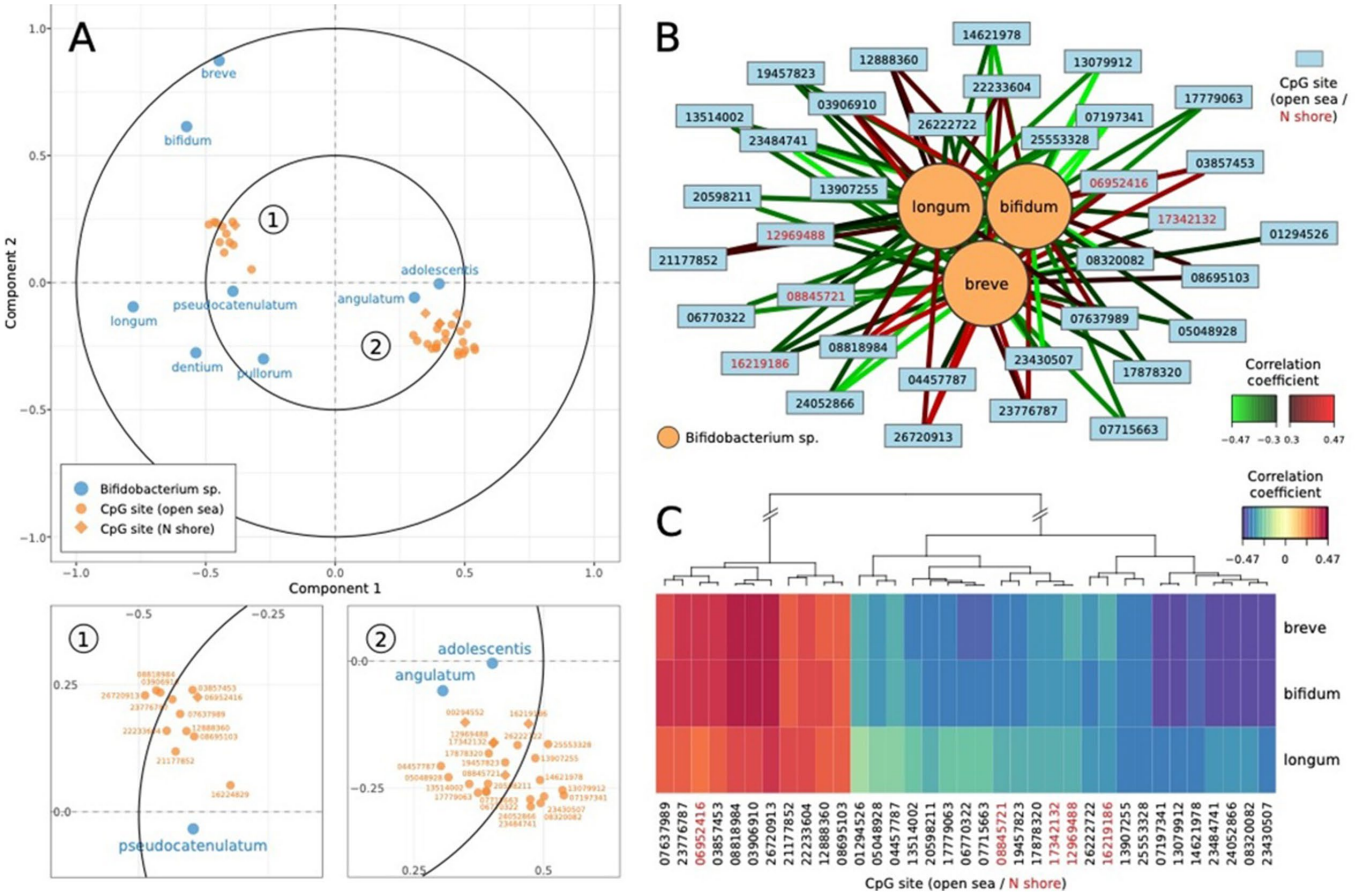
Relationships between *NR3C1* CpG methylation sites and *Bifidobacterium* species. **(A)** The correlation circle plot reveals distinct associations between HPA-related CpG sites and *Bifidobacterium* strains, with microbial species such as *bifidum* and *breve* showing stronger correlations with principal components compared to others like *pullorum* and *longum*. **(B)** The relevance network highlights the complex interplay between CpG sites and *Bifidobacterium* species, with *bifidum* exhibiting multiple significant positive and negative correlations. **(C)** The heatmap illustrates the correlation patterns between CpG sites and *Bifidobacterium* species, with distinct clusters of CpG sites sharing similar positive and negative patterns across species.

Regularized canonical correlation analysis (rCCA) was performed to investigate the relationships between microbial species (dataset X) and CpG sites (dataset Y), focusing on their contributions to the first two canonical components (full rCCA loading values are included in Supplementary Table 4). The canonical correlations between the two datasets were strong for the first (r = 0.64; permuted *p* < 0.0001; Supplementary Figure 6) and second canonical variates (r = 0.56; permuted *p* = 0.7676). For canonical variates 1, microbial features such as *angulatum* (0.36), *catenulatum* (0.18), and *adolescentis* (0.17) displayed strong positive loadings, indicating their significant role in explaining the variation captured by this dimension. Conversely, *dentium* (−0.52) and *pullorum* (−0.25) had strong negative loadings, suggesting opposing contributions. Similarly, CpG sites such as cg06521673 (0.61), cg03746860 (0.56), and cg19641581 (0.56) were among the most influential positive contributors, while cg24801588 (−0.70), cg05048928 (−0.77), and cg14438279 (−0.63) had significant negative contributions. For canonical variate 2, microbial features *breve* (0.10) and *bifidum* (0.09) positively contributed, whereas *dentium* (−0.59) and *pullorum* (−0.43) exhibited strong negative associations. CpG sites such as cg15740681 (0.82) and cg23273257 (0.64) strongly influenced canonical variate 2 positively, while cg25535999 (−1.18), cg19176661 (−0.78), and cg19645279 (−0.74) negatively impacted this dimension. These findings suggest distinct sets of microbial species and CpG sites driving variability in the canonical components, with positive loadings reflecting coordinated biological processes and negative loadings indicating opposing relationships. Component 1 was dominated by microbial contributions, particularly *angulatum* and *catenulatum*, while Component 2 was more heavily influenced by CpG methylation sites such as cg15740681 and cg23273257. Together, these results highlight the complex interplay between the microbiome and epigenome, offering valuable insights into potential shared pathways or mechanisms underlying these associations. Figure 2 represents the categorization of the relationship between *NR3C1* CpG methylation sites and *Bifidobacterium* species.

## 4 Discussion

This is the first study to investigate gut microbiome composition in relation to HPA gene DNA methylation. We assessed the relationships between a probiotic-folate-producing genus, *Bifidobacterium*, and DNA methylation of key HPA genes, including *NR3C1*, *FKBP5*, *AVP*, *CRH, CRHR1*, and *CRHR2*. Results revealed robust associations between DNA methylation at CpG sites across the gene body for all HPA genes examined. Additionally, the abundance of *Bifidobacterium* species does not predict global DNA methylation, indicating that its impact on DNA methylation is more specifically linked to cortisol-related genes rather than overall DNA methylation levels. Finally, association-based mediation analysis revealed that factors beyond age partially drive these associations.

We further probed associations between *Bifidobacterium* abundance and *NR3C1* DNA methylation through a multi-omic regularized canonical correlation model. Findings revealed a complex interplay between *Bifidobacterium* and HPA-related CpG methylation sites, underscoring shared biological pathways linking the microbiome and epigenome related to stress and anxiety. Notably, canonical variate 1 was heavily influenced by *Bifidobacterium* species such as *angulatum*, *catenulatum*, and *adolescentis*, alongside CpG sites like cg06521673 and cg03746860, which positively loaded onto this dimension. This alignment may point to coordinated biological processes where specific microbial communities influence methylation patterns in a concerted manner. Conversely, negative loadings for features such as *dentium* and cg24801588 suggest opposing roles within these interactions.

Similarly, canonical variate 2 demonstrated distinct patterns, with CpG sites like cg15740681 and cg23273257 exerting strong positive influences, while microbial species such as *dentium* and CpG sites like cg25535999 contributed negatively. DNA methylation at open sea CpG sites can have functional regulatory consequences. Using mSTARR-seq, Lea et al. (2018) identified thousands of methylation-dependent enhancer elements in open sea regions that directly modulate gene expression. Additionally, synthetic methylation studies in yeast and human cell lines show that methylation in these regions can alter chromatin accessibility and transcription factor binding (Lea et al., 2018; Shipony et al., 2020). These results suggest that while microbial communities dominate some dimensions of this interplay, others are primarily driven by epigenetic variations. Taken together, these findings offer valuable insights into how microbial and epigenetic interactions may converge in influencing stress physiology and anxiety phenotypes, potentially providing a framework for understanding shared mechanisms underlying mental health.

Considering the established links between HPA dysregulation (Murphy et al., 2022), epigenetic mechanisms (Keverne and Binder, 2020), and microbiome composition (Kelly et al., 2015) in psychiatric disorders, the epigenetic modifying potentials of microbiome metabolites are not well studied (Stilling et al., 2014). While the current study cannot ascertain the direction of effects or an associative relationship, it provides evidence that levels of the probiotic genus, *Bifidobacterium* spp., may influence DNA methylation of HPA genes. If such a pathway can be established in future mechanistic studies, microbiome-modifying exposures such as diet, exercise, and probiotics could be explored specifically as epigenetic-targeting psychiatric treatments. Slight alterations in DNAm are known to impact genetic regulation and downstream function (Jones, 2012). For example, our lab previously demonstrated that peripheral DNA methylation of HPA-related genes is predictive of the diurnal cortisol slope (Lewis et al., 2021). Importantly, gut microbiota composition may impact the epigenome widely (Kumar et al., 2014). As such, associations between gut microbiome and DNA methylation found is this study suggest the potential pathway that gut microbiome composition impacts HPA-related gene regulation and downstream physiology. However, more research assessing this pathway is necessary.

Microbiome effects on the host epigenome are a likely pathway in many disease states (Gillman and Blaisdell, 2018; Aryee et al., 2014). For instance, the role of microbial metabolites in mediating the well-established link between diet and the epigenome has been extensively studied over the past decade (McIver et al., 2018; Beghini et al., 2021; Murphy et al., 2022). Changes in microbial composition influence epigenetic patterns underlying metabolic syndrome (Keverne and Binder, 2020). The gut microbiota plays a crucial role in colorectal carcinogenesis by either directly or indirectly affecting local epigenetics (Stilling et al., 2014). Various microbial manipulation therapies for lung cancer demonstrate impressive results through modulating epigenetic homeostasis of the lung and the epigenetic aberrations in lung carcinogenesis (Barton et al., 2019). Microbiome transplant therapies have also demonstrated changes in the behavior and epigenetics of autistic individuals (Nabais et al., 2023; Van Puyvelde et al., 2023). Other studies have focused on the immediate vicinity of the gut microbiome and have found influences on the host’s intestinal epigenetics and local homeostasis (El-Sayed et al., n.d.). Our findings add to this growing body of evidence, suggesting that associations between microbiome composition, cortisol levels, and psychiatric symptoms may be partially driven by microbe-derived folate influencing epigenetic regulation of HPA-axis genes.

A limitation of this study is that we were unable to control for dietary intake, which impacts microbiome composition. Given the harmonized protocols of the ECHO consortium, potential biases from individual cohort designs are likely minimized, though we acknowledge this as a limitation to this work. It should also be acknowledged that limiting the global analysis to the top 50% most variable CpG sites may exclude low-variance CpG sites that could still be biologically important, particularly those involved in stable regulatory functions or developmentally constrained pathways. We also did not include a cell composition variable, an important covariate in epigenetic studies, due to the high collinearity with all of the DNA methylation principal components. However, others have shown that including cell-type heterogeneity adjustment does not always improve the analysis (Barton et al., 2019; Nabais et al., 2023). Since this study only assessed *Bifidobacterium,* future studies could include the relative abundance of all known folate producers and other microbiome-produced metabolites that affect DNA methylation, such as betaine and choline (Van Puyvelde et al., 2023). Future studies could include cortisol output to further elaborate on downstream effects of the epigenome on host physiology.

In conclusion, microbiome metabolites may influence stress physiology through altering the host epigenome (El-Sayed et al., n.d.). The interaction between the developing gut microbiome and epigenetic processes may play a critical role in brain health throughout development (Kaur et al., 2021; Alam et al., 2017). This study highlights biologically plausible associations between *Bifidobacterium* and HPA gene DNA methylation in a healthy pediatric cohort. While the directionality of effects between the microbiome and epigenome remains unclear, evidence suggests a bidirectional relationship between these dynamic systems. Clarifying this association is crucial to understanding how interventions targeting the microbiome, or epigenome, might mitigate psychiatric vulnerability. Future longitudinal studies are needed to more directly assess temporal order and potential causality between microbiome composition and host DNA methylation. Additional investigations should explore an integrated analysis between the complete gut microbiome and epigenome-wide DNA methylation. A more comprehensive exploration of mental health etiology, along with the intricate pathways connecting environmental exposures to biological processes, could usher in a new era of understanding and prevention strategies.

## Data Availability

The datasets presented in this study are publicly available. This data can be found here: https://www.ncbi.nlm.nih.gov, accession number PRJNA695570.
