# Supplementary figures and images for: Associations between relative abundances of Bifidobacterium species in the gut and DNA methylation of cortisol-related genes in a pediatric population
Source: Front Microbiol. 2025 Sep 22;16:1558809. doi: 10.3389/fmicb.2025.1558809 (PMC12498159; doi:10.3389/fmicb.2025.1558809)

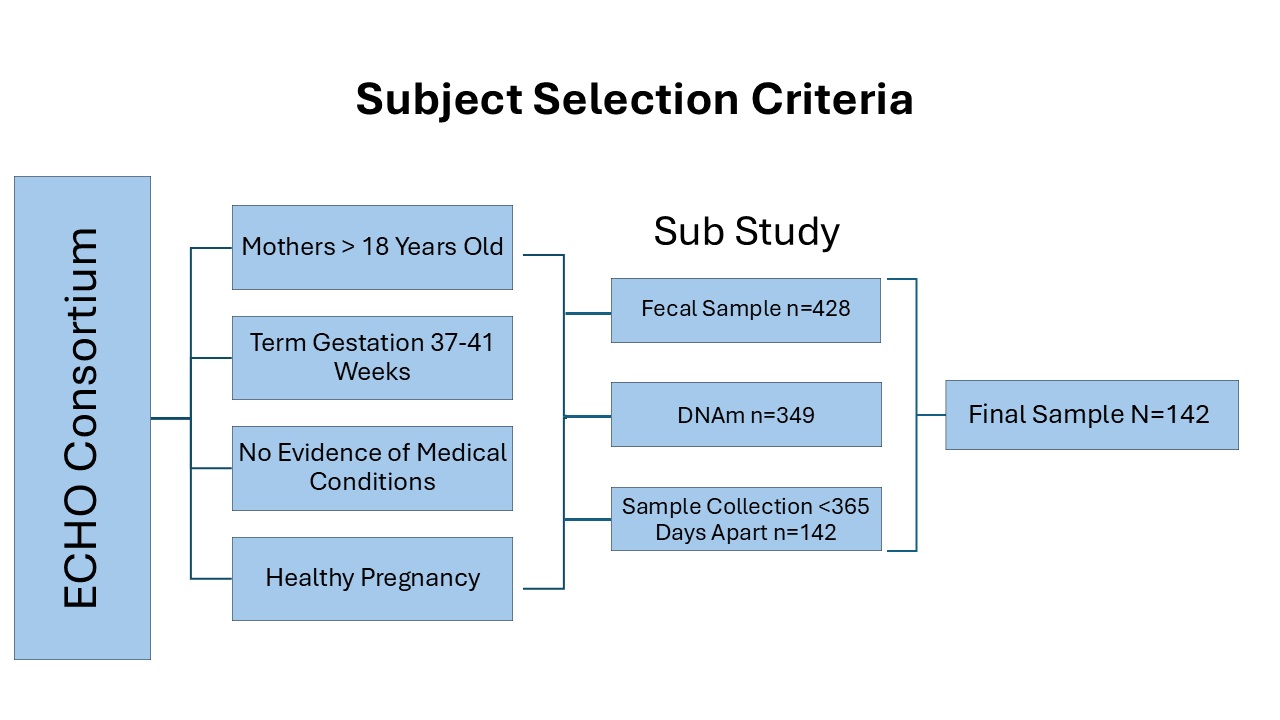

Supplement: Supplementary file 1 [file Image_1.jpeg]

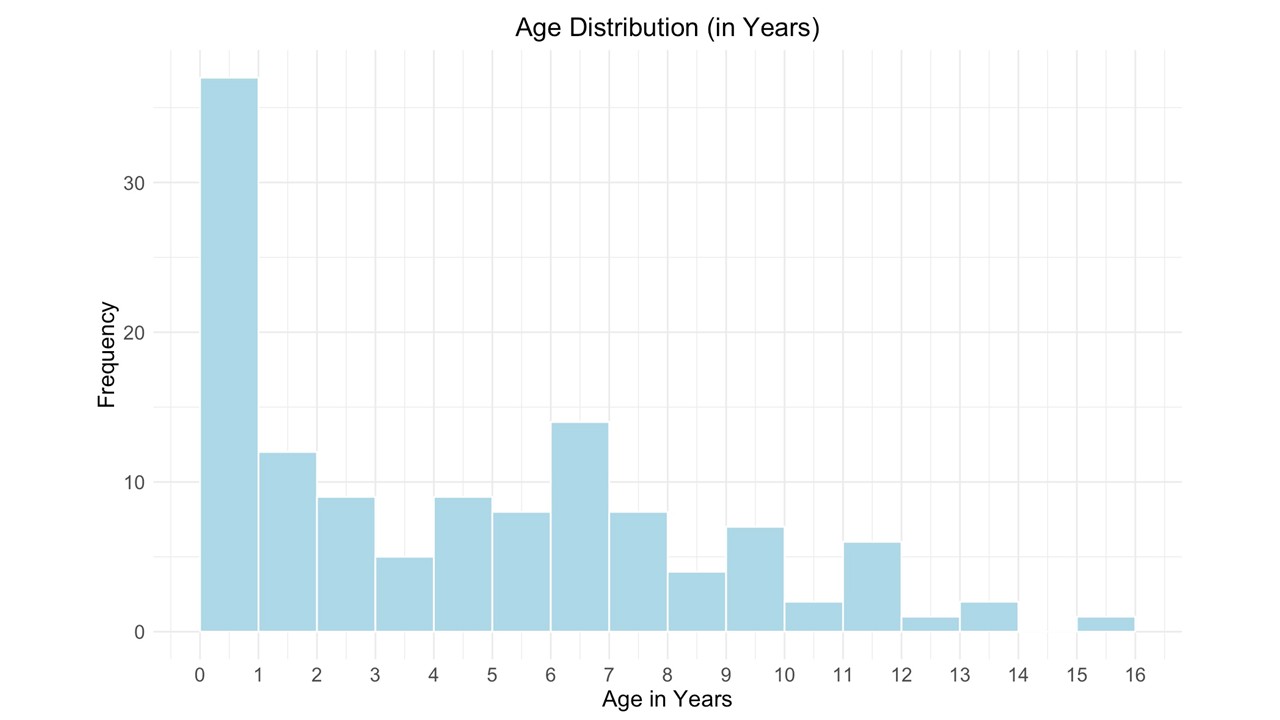

Supplement: Supplementary file 2 [file Image_2.jpeg]

**Scree plot**

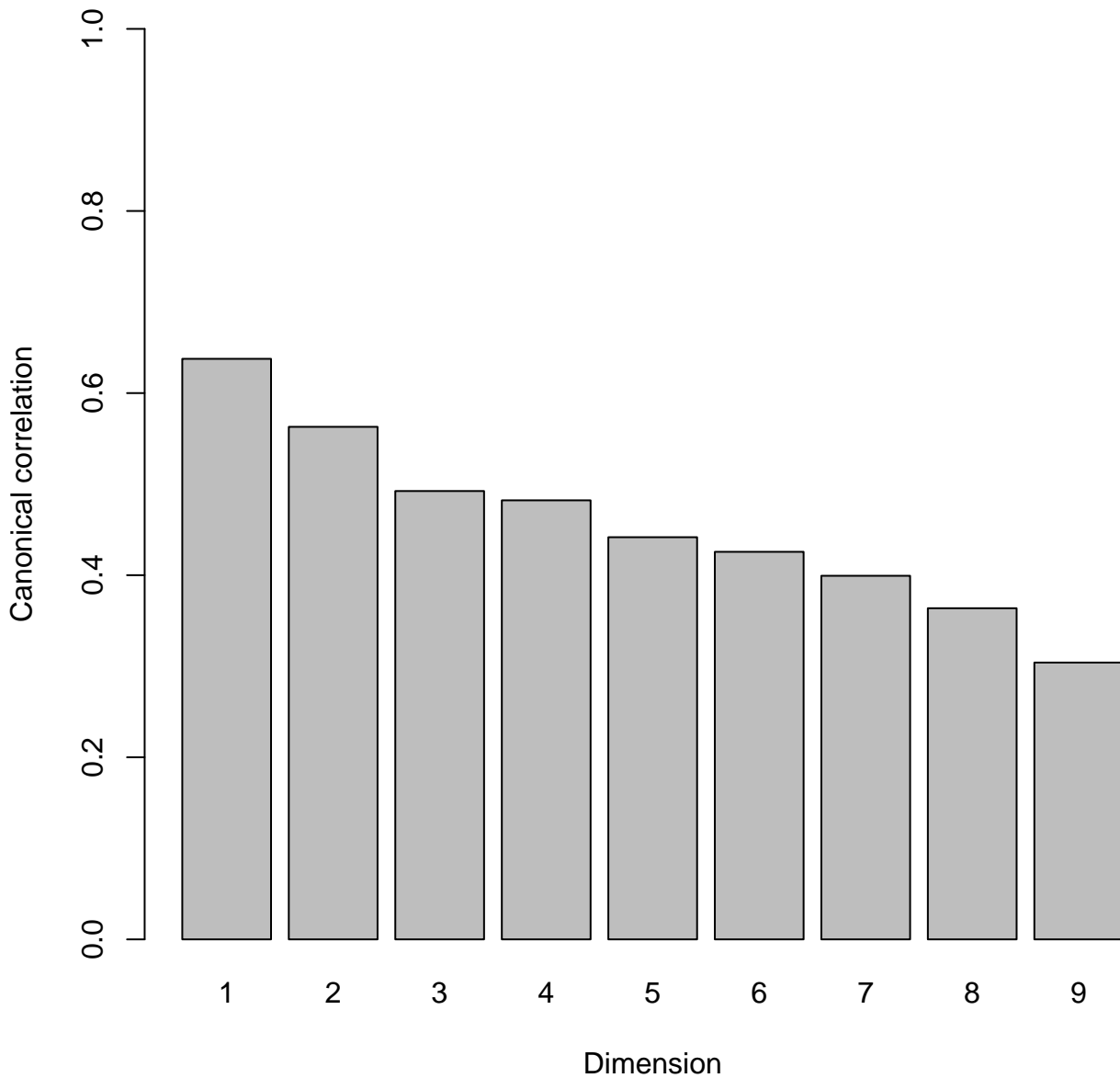

Supplement: Supplementary file 3 [file Image_3.pdf]

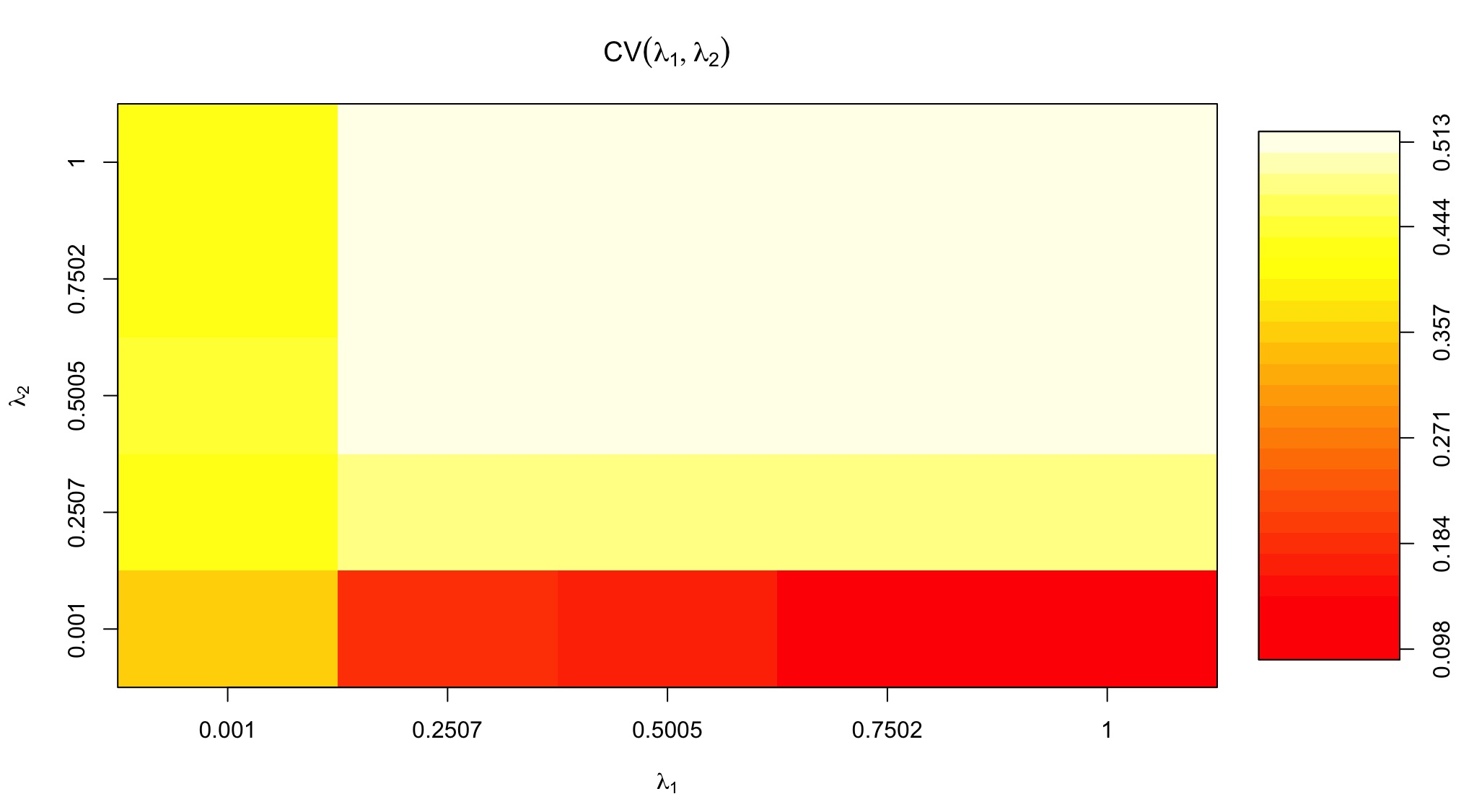

Supplement: Supplementary file 4 [file Image_4.jpeg]

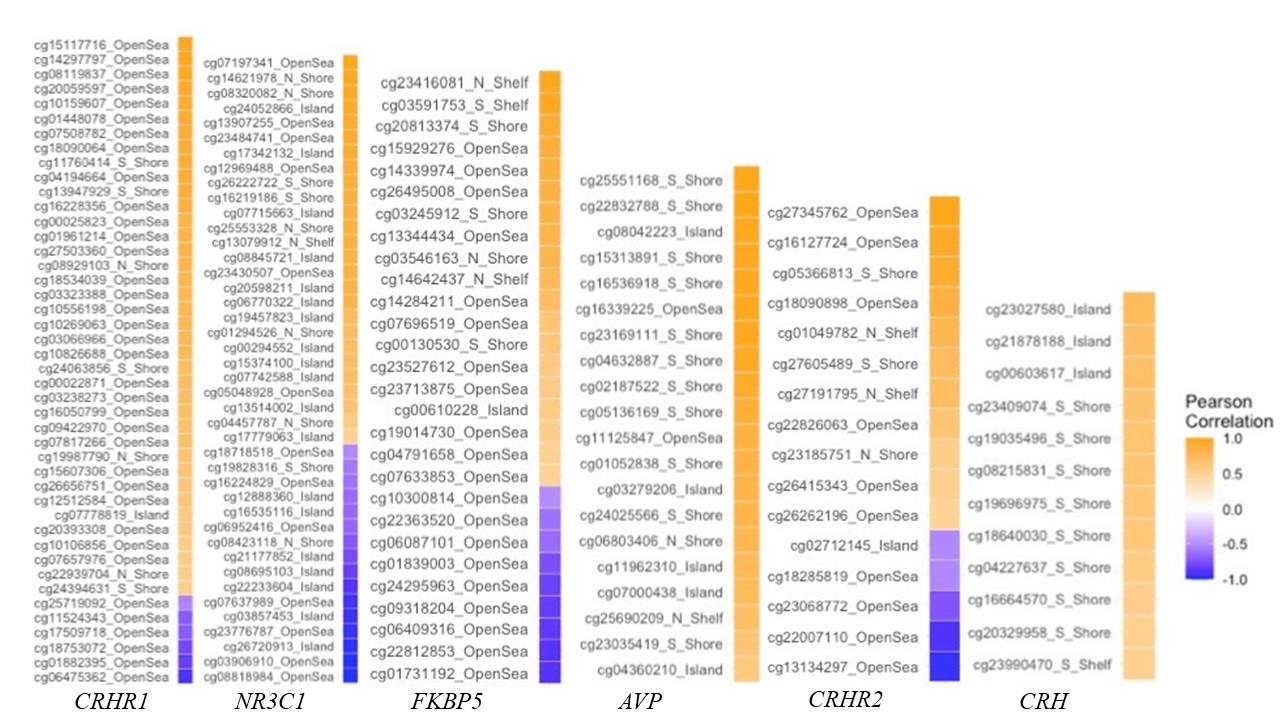

Supplement: Supplementary file 5 [file Image_5.jpeg]

# Permutation Null for rCCA (comp1)

$p = 9.999e-05$

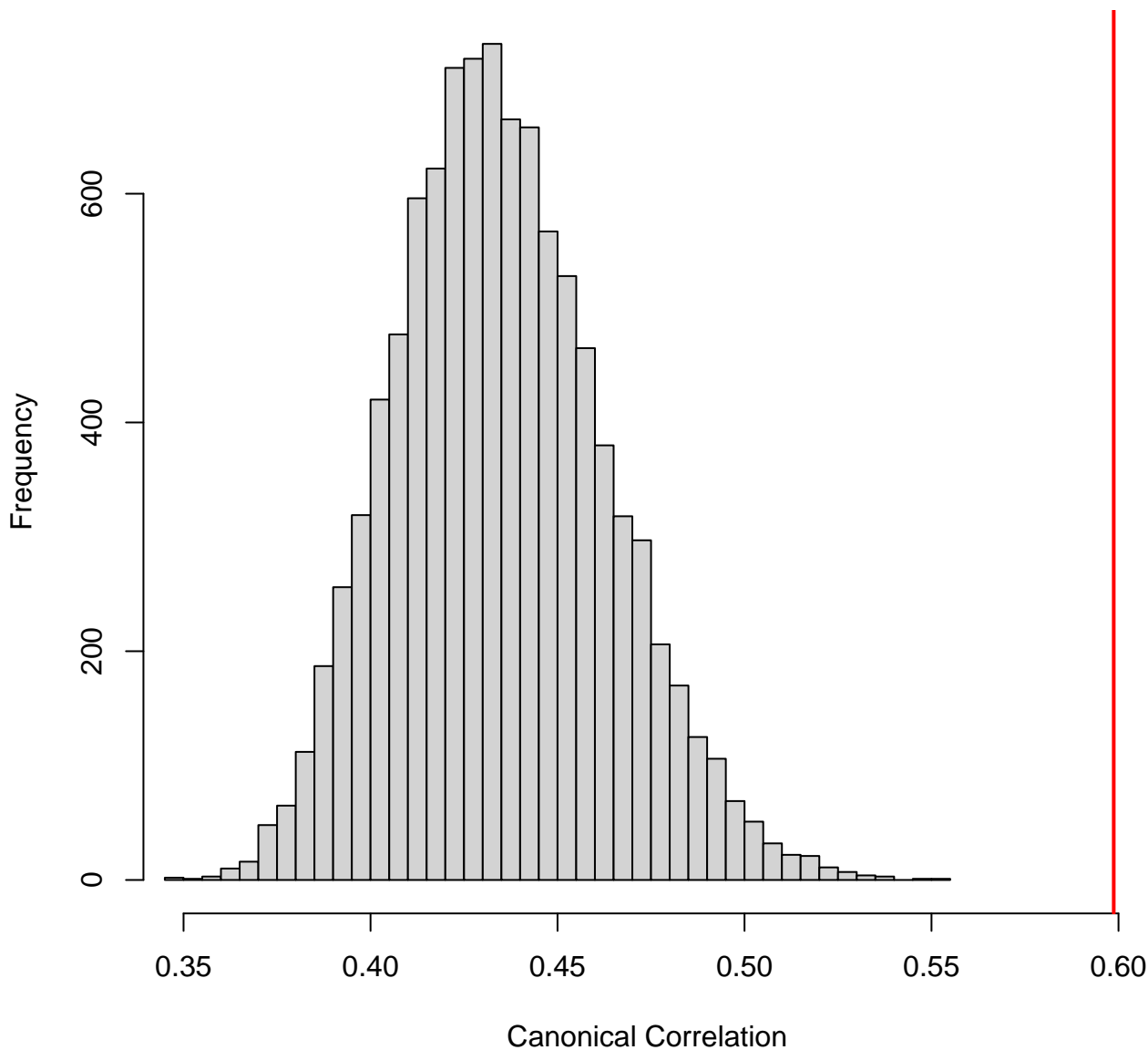

Supplement: Supplementary file 6 [file Image_6.pdf]
