# Supplementary material for: Associations between relative abundances of Bifidobacterium species in the gut and DNA methylation of cortisol-related genes in a pediatric population
Source: Front Microbiol. 2025 Sep 22;16:1558809. doi: 10.3389/fmicb.2025.1558809 (PMC12498159; doi:10.3389/fmicb.2025.1558809)
Supplement: Supplementary file 7 [file Supplementary_file_1.docx]

Supplementary Material

| **Supp Table 1. Bifidobacterium Species included in the CCA Model**  Summary of *bifidobacterium* sum composite and species used in rCCA | | | | | | | |
| --- | --- | --- | --- | --- | --- | --- | --- |
| ***Bifidobacterium* Sum Composite** | | | | | | ***Bifidobacterium* Species Used in rCCA** | |
| Bifidobacterium_adolescentis | | | | | | Bifidobacterium_longum | |
| Bifidobacterium_angulatum | | | | | | Bifidobacterium_pseudocatenulatum | |
| Bifidobacterium_animalis | | | | | | Bifidobacterium_adolescentis | |
| Bifidobacterium_bifidum | | | | | | Bifidobacterium_bifidum | |
| Bifidobacterium_breve | | | | | | Bifidobacterium_catenulatum | |
| Bifidobacterium_catenulatum | | | | | | Bifidobacterium_breve | |
| Bifidobacterium_dentium | | | | | | Bifidobacterium_angulatum | |
| Bifidobacterium_gallinarum | | | | | | Bifidobacterium_pullorum | |
| Bifidobacterium_kashiwanohense | | | | | | Bifidobacterium_dentium | |
| Bifidobacterium_longum | | | | | |  | |
| Bifidobacterium_moukalabense | | | | | |  | |
| Bifidobacterium_pseudocatenulatum | | | | | |  | |
| Bifidobacterium_pseudolongum | | | | | |  | |
| Bifidobacterium_pullorum | | | | | |  | |
| Bifidobacterium_ruminantium | | | | | |  | |
| Bifidobacterium_saeculare | | | | | |  | |
| Bifidobacterium_scardovii | | | | | |  | |
| **Supp Table 2. Location and loading values for DNA methylation PCA first principal component**  CpG, location, and loading value for DNA methylation PCA of each cortisol-related gene | | | | |  |  |  |
| *NR3C1* | | | | |  |  |  |
| CpG | Location | | r | |  |  |  |
| cg07197341 | OpenSea | | 0.97 | |  |  |  |
| cg14621978 | N_Shore | | 0.97 | |  |  |  |
| cg08320082 | N_Shore | | 0.96 | |  |  |  |
| cg24052866 | Island | | 0.93 | |  |  |  |
| cg13907255 | OpenSea | | 0.92 | |  |  |  |
| cg23484741 | OpenSea | | 0.91 | |  |  |  |
| cg17342132 | Island | | 0.91 | |  |  |  |
| cg12969488 | OpenSea | | 0.89 | |  |  |  |
| cg26222722 | S_Shore | | 0.88 | |  |  |  |
| cg16219186 | S_Shore | | 0.87 | |  |  |  |
| cg07715663 | Island | | 0.86 | |  |  |  |
| cg25553328 | N_Shore | | 0.86 | |  |  |  |
| cg13079912 | N_Shelf | | 0.86 | |  |  |  |
| cg08845721 | Island | | 0.84 | |  |  |  |
| cg23430507 | OpenSea | | 0.84 | |  |  |  |
| cg20598211 | Island | | 0.84 | |  |  |  |
| cg06770322 | Island | | 0.83 | |  |  |  |
| cg08695103 | Island | | -0.83 | |  |  |  |
| cg22233604 | Island | | -0.89 | |  |  |  |
| cg07637989 | OpenSea | | -0.93 | |  |  |  |
| cg03857453 | Island | | -0.95 | |  |  |  |
| cg23776787 | OpenSea | | -0.95 | |  |  |  |
| cg26720913 | Island | | -0.97 | |  |  |  |
| cg03906910 | OpenSea | | -0.98 | |  |  |  |
| cg08818984 | OpenSea | | -0.98 | |  |  |  |
| *FKBP5* | | | | |  |  |  |
| cg23416081 | N_Shelf | | 0.96 | |  |  |  |
| cg03591753 | S_Shelf | | 0.96 | |  |  |  |
| cg20813374 | S_Shore | | 0.91 | |  |  |  |
| cg15929276 | OpenSea | | 0.89 | |  |  |  |
| cg14339974 | OpenSea | | 0.87 | |  |  |  |
| cg26495008 | OpenSea | | 0.84 | |  |  |  |
| cg03245912 | S_Shore | | 0.83 | |  |  |  |
| cg13344434 | OpenSea | | 0.82 | |  |  |  |
| cg03546163 | N_Shore | | 0.80 | |  |  |  |
| cg24295963 | OpenSea | | -0.82 | |  |  |  |
| cg09318204 | OpenSea | | -0.84 | |  |  |  |
| cg06409316 | OpenSea | | -0.85 | |  |  |  |
| cg22812853 | OpenSea | | -0.88 | |  |  |  |
| cg01731192 | OpenSea | | -0.88 | |  |  |  |
| *AVP* | | | | |  |  |  |
| cg25551168 | S_Shore | | 0.97 | |  |  |  |
| cg22832788 | S_Shore | | 0.97 | |  |  |  |
| cg08042223 | Island | | 0.96 | |  |  |  |
| cg15313891 | S_Shore | | 0.96 | |  |  |  |
| cg16536918 | S_Shore | | 0.96 | |  |  |  |
| cg16339225 | OpenSea | | 0.96 | |  |  |  |
| cg23169111 | S_Shore | | 0.95 | |  |  |  |
| cg04632887 | S_Shore | | 0.94 | |  |  |  |
| cg02187522 | S_Shore | | 0.92 | |  |  |  |
| cg05136169 | S_Shore | | 0.89 | |  |  |  |
| cg11125847 | OpenSea | | 0.85 | |  |  |  |
| cg01052838 | S_Shore | | 0.84 | |  |  |  |
| cg03279206 | Island | | 0.83 | |  |  |  |
| cg24025566 | S_Shore | | 0.82 | |  |  |  |
| *CRH* | | | | |  |  |  |
| cg00603617 | Island | | 0.69 | |  |  |  |
| cg04227637 | S_Shore | | 0.56 | |  |  |  |
| cg08215831 | S_Shore | | 0.64 | |  |  |  |
| cg16664570 | S_Shore | | 0.52 | |  |  |  |
| cg18640030 | S_Shore | | 0.61 | |  |  |  |
| cg19035496 | S_Shore | | 0.65 | |  |  |  |
| cg19696975 | S_Shore | | 0.62 | |  |  |  |
| cg20329958 | S_Shore | | 0.50 | |  |  |  |
| cg21878188 | Island | | 0.71 | |  |  |  |
| cg23027580 | Island | | 0.75 | |  |  |  |
| cg23409074 | S_Shore | | 0.67 | |  |  |  |
| cg23990470 | S_Shelf | | 0.50 | |  |  |  |
| *CRHR1* | | | | |  |  |  |
| cg15117716 | OpenSea | | 0.97 | |  |  |  |
| cg14297797 | OpenSea | | 0.97 | |  |  |  |
| cg08119837 | OpenSea | | 0.96 | |  |  |  |
| cg20059597 | OpenSea | | 0.94 | |  |  |  |
| cg10159607 | OpenSea | | 0.92 | |  |  |  |
| cg01448078 | OpenSea | | 0.92 | |  |  |  |
| cg07508782 | OpenSea | | 0.90 | |  |  |  |
| cg18090064 | OpenSea | | 0.90 | |  |  |  |
| cg11760414 | S_Shore | | 0.90 | |  |  |  |
| cg04194664 | OpenSea | | 0.90 | |  |  |  |
| cg13947929 | S_Shore | | 0.89 | |  |  |  |
| cg16228356 | OpenSea | | 0.87 | |  |  |  |
| cg00025823 | OpenSea | | 0.85 | |  |  |  |
| cg01961214 | OpenSea | | 0.84 | |  |  |  |
| cg27503360 | OpenSea | | 0.83 | |  |  |  |
| cg08929103 | N_Shore | | 0.82 | |  |  |  |
| cg18534039 | OpenSea | | 0.81 | |  |  |  |
| *CRHR2* | | | | |  |  |  |
| cg27345762 | OpenSea | | 0.97 | |  |  |  |
| cg16127724 | OpenSea | | 0.93 | |  |  |  |
| cg05366813 | S_Shore | | 0.90 | |  |  |  |
| cg18090898 | OpenSea | | 0.85 | |  |  |  |
| cg01049782 | N_Shelf | | 0.78 | |  |  |  |
| cg27605489 | S_Shore | | 0.73 | |  |  |  |
| cg27191795 | N_Shelf | | 0.71 | |  |  |  |
| cg22826063 | OpenSea | | 0.64 | |  |  |  |
| cg23185751 | N_Shore | | 0.53 | |  |  |  |
| cg26415343 | OpenSea | | 0.52 | |  |  |  |
| cg26262196 | OpenSea | | 0.50 | |  |  |  |
| cg02712145 | Island | | -0.52 | |  |  |  |
| cg18285819 | OpenSea | | -0.52 | |  |  |  |
| cg23068772 | OpenSea | | -0.74 | |  |  |  |
| cg22007110 | OpenSea | | -0.90 | |  |  |  |
| cg13134297 | OpenSea | | -0.95 | |  |  |  |
| \| **Supp Table 3. HPA gene DNA methylation and *Bifidobacterium* Mediation Model Results** \| \| \| \| \| \|  \|  \|  \|  \|  \| \| --- \| --- \| --- \| --- \| --- \| --- \| --- \| --- \| --- \| --- \| --- \| \| Results from bifidobacterium mediation model for cortisol-related gene DNA methylation \| \| \| \| \| \| \| ***Bifidobacterium*** \| \| \| \| \| \| \| \| \| \| \| \|  \| **PC1** \| \| \| \| \| \| **PC2** \| \| \| \| \| **Gene** \| **Effect** \| \| **Estimate** \| **95% CI Lower** \| **95% CI Upper** \| **p-value** \| **Estimate** \| **95% CI Lower** \| **95% CI Upper** \| **p-value** \| \| *NR3C1* \| ACME (Indirect) \| \| -0.26 \| -0.35 \| -0.2 \| <2e-16 \| 0.08 \| 0.02 \| 0.15 \| 0.01 \| \| ADE (Direct) \| \| -0.17 \| -0.26 \| -0.09 \| <2e-16 \| -0.02 \| -0.15 \| 0.11 \| 0.7 \| \| Total Effect \| \| -0.43 \| -0.53 \| -0.34 \| <2e-16 \| 0.06 \| -0.05 \| 0.18 \| 0.35 \| \| Proportion Mediated \| \| 0.6 \| 0.46 \| 0.77 \| <2e-16 \| 1.4 \| -15.52 \| 13.94 \| 0.36 \| \| *FKBP5* \| ACME (Indirect) \| \| 0.26 \| 0.18 \| 0.35 \| <2e-16 \| -0.02 \| -0.08 \| 0.04 \| 0.57 \| \| ADE (Direct) \| \| 0.2 \| 0.1 \| 0.3 \| <2e-16 \| -0.08 \| -0.19 \| 0.04 \| 0.18 \| \| Total Effect \| \| 0.45 \| 0.35 \| 0.58 \| <2e-16 \| -0.1 \| -0.19 \| 0.01 \| 0.09 \| \| Proportion Mediated \| \| 0.57 \| 0.42 \| 0.74 \| <2e-16 \| 0.18 \| -0.93 \| 2.28 \| 0.59 \| \| *AVP* \| ACME (Indirect) \| \| 0.25 \| 0.19 \| 0.35 \| <2e-16 \| -0.04 \| -0.11 \| 0.02 \| 0.2 \| \| ADE (Direct) \| \| 0.19 \| 0.09 \| 0.29 \| <2e-16 \| -0.11 \| -0.26 \| 0.05 \| 0.17 \| \| Total Effect \| \| 0.44 \| 0.34 \| 0.57 \| <2e-16 \| -0.15 \| -0.28 \| 0.01 \| 0.07 \| \| Proportion Mediated \| \| 0.58 \| 0.43 \| 0.77 \| <2e-16 \| 0.24 \| -0.8 \| 1.71 \| 0.24 \| \| *CRH* \| ACME (Indirect) \| \| 0.06 \| -0.01 \| 0.14 \| 0.08 \| 0.22 \| 0.16 \| 0.31 \| <2e-16 \| \| ADE (Direct) \| \| -0.14 \| -0.26 \| -0.01 \| 0.03 \| 0.23 \| 0.1 \| 0.35 \| <2e-16 \| \| Total Effect \| \| -0.08 \| -0.19 \| 0.04 \| 0.2 \| 0.45 \| 0.34 \| 0.56 \| <2e-16 \| \| Proportion Mediated \| \| -0.74 \| -5.99 \| 11.78 \| 0.27 \| 0.5 \| 0.33 \| 0.74 \| <2e-16 \| \| *CRHR1* \| ACME (Indirect) \| \| -0.24 \| -0.33 \| -0.17 \| <2e-16 \| 0.07 \| 0 \| 0.14 \| 0.05 \| \| ADE (Direct) \| \| -0.18 \| -0.26 \| -0.1 \| <2e-16 \| 0.06 \| -0.07 \| 0.21 \| 0.41 \| \| Total Effect \| \| -0.42 \| -0.52 \| -0.33 \| <2e-16 \| 0.13 \| 0.01 \| 0.27 \| 0.03 \| \| Proportion Mediated \| \| 0.57 \| 0.44 \| 0.72 \| <2e-16 \| 0.56 \| -0.1 \| 3.48 \| 0.07 \| \| *CRHR2* \| \| ACME (Indirect) \| -0.25 \| -0.34 \| -0.17 \| <2e-16 \| 0 \| -0.07 \| 0.06 \| 0.94 \| \| ADE (Direct) \| -0.15 \| -0.25 \| -0.07 \| <2e-16 \| -0.02 \| -0.15 \| 0.12 \| 0.79 \| \| Total Effect \| -0.4 \| -0.51 \| -0.3 \| <2e-16 \| -0.02 \| -0.14 \| 0.1 \| 0.71 \| \| Proportion Mediated \| 0.62 \| 0.46 \| 0.8 \| <2e-16 \| 0.07 \| -6.57 \| 5.07 \| 0.99 \|   **Supp Table 4. rCCA loading values**  *Bifidobacterium species and relevant CpG sites for rCCA loading values* | | | | | | |  |
| **Species** | | **1** | | **2** | | |  |
| *dentium* | | -0.5162795 | | -0.5865232 | | |  |
| *pullorum* | | -0.2513151 | | -0.4303921 | | |  |
| *longum* | | -0.0775182 | | -0.0224267 | | |  |
| *bifidum* | | -0.0613539 | | 0.08775504 | | |  |
| *breve* | | -0.0399022 | | 0.10309763 | | |  |
| *pseudocatenulatum* | | -0.0397859 | | -0.0036199 | | |  |
| *adolescentis* | | 0.16844666 | | 0.02021967 | | |  |
| *catenulatum* | | 0.17962143 | | -0.0763947 | | |  |
| *angulatum* | | 0.35697467 | | -0.1332335 | | |  |
| **CpG sites** | |  | |  | | |  |
| cg21177852OpenSea | | -0.3942616 | | -0.1945519 | | |  |
| cg15374100OpenSea | | -0.3333189 | | 0.33102419 | | |  |
| cg15740681OpenSea | | -0.0594358 | | 0.82214983 | | |  |
| cg23273257OpenSea | | -0.1073782 | | 0.6398758 | | |  |
| cg26081259OpenSea | | 0.04534053 | | 0.30006248 | | |  |
| cg13514002OpenSea | | 0.16465816 | | -0.1834205 | | |  |
| cg24801588OpenSea | | -0.6992809 | | -0.8662397 | | |  |
| cg00407401OpenSea | | -0.00882 | | -0.2752171 | | |  |
| cg19457823OpenSea | | 0.14543214 | | -0.1518445 | | |  |
| cg12741214OpenSea | | -0.4038968 | | -0.393129 | | |  |
| cg04457787OpenSea | | 0.30921564 | | -0.1180947 | | |  |
| cg20728768OpenSea | | 0.11791552 | | 0.1675821 | | |  |
| cg25708981OpenSea | | 0.01838977 | | 0.050487 | | |  |
| cg19645279OpenSea | | -0.3241279 | | -0.7348054 | | |  |
| cg19176661OpenSea | | -0.4225496 | | -0.7753961 | | |  |
| cg07715663OpenSea | | -0.2935346 | | -0.2817997 | | |  |
| cg24052866OpenSea | | 0.08535665 | | -0.1755195 | | |  |
| cg22233604OpenSea | | -0.0113963 | | -0.2929369 | | |  |
| cg03857453OpenSea | | 0.21920395 | | 0.03339411 | | |  |
| cg15115787OpenSea | | 0.0400764 | | -0.0919471 | | |  |
| cg12888360OpenSea | | 0.00208003 | | -0.0263071 | | |  |
| cg08695103OpenSea | | 0.1877689 | | -0.0587022 | | |  |
| cg14621978OpenSea | | 0.09658488 | | -0.0261181 | | |  |
| cg18484679OpenSea | | -0.0969505 | | 0.14571311 | | |  |
| cg16586394OpenSea | | 0.18976818 | | 0.17532554 | | |  |
| cg25535999OpenSea | | -0.2740438 | | -1.1813729 | | |  |
| cg03746860OpenSea | | 0.55488971 | | -0.3566443 | | |  |
| cg20598211OpenSea | | -0.2535202 | | -0.4100558 | | |  |
| cg05483455OpenSea | | -0.0222356 | | -0.4065891 | | |  |
| cg16594263OpenSea | | 0.13300942 | | -0.8975391 | | |  |
| cg16535116OpenSea | | 0.07056088 | | 0.13301716 | | |  |
| cg05900547OpenSea | | 0.09782545 | | -0.039595 | | |  |
| cg19432243OpenSea | | 0.29327215 | | -0.1243491 | | |  |
| cg19820298OpenSea | | 0.07911947 | | -0.1467218 | | |  |
| cg27107893OpenSea | | -0.1022033 | | -0.0843936 | | |  |
| cg06613263N_Shelf | | 0.20552988 | | -0.2855971 | | |  |
| cg17342132N_Shore | | 0.09618218 | | 0.0202124 | | |  |
| cg07742588N_Shore | | -0.098931 | | 0.40589869 | | |  |
| cg00294552N_Shore | | 0.07324702 | | -0.0266565 | | |  |
| cg16219186N_Shore | | 0.05022031 | | 0.41419542 | | |  |
| cg08845721N_Shore | | 0.1772182 | | -0.1148607 | | |  |
| cg12969488N_Shore | | -0.3132103 | | -0.1050838 | | |  |
| cg07733851N_Shore | | 0.20865758 | | 0.60426749 | | |  |
| cg18998365N_Shore | | -0.1531644 | | 0.17855727 | | |  |
| cg27122725N_Shore | | -0.2334812 | | 0.0303888 | | |  |
| cg06952416N_Shore | | -0.1258449 | | -0.2989298 | | |  |
| cg06521673Island | | 0.6070479 | | -0.0386798 | | |  |
| cg17617527Island | | -0.1746103 | | 0.19317724 | | |  |
| cg20753294Island | | -0.0926425 | | -0.2811269 | | |  |
| cg18146873Island | | 0.34536479 | | 0.02842815 | | |  |
| cg00629244Island | | -0.0485786 | | -0.0883446 | | |  |
| cg11152298Island | | -1.0405413 | | -0.1951686 | | |  |
| cg18019515Island | | -0.2210042 | | -0.1226089 | | |  |
| cg17860381Island | | 0.27710653 | | -0.4239218 | | |  |
| cg04111177Island | | 0.3952946 | | -0.2228168 | | |  |
| cg15910486Island | | -0.0199622 | | 0.43654537 | | |  |
| cg15645634Island | | -0.0288884 | | 0.25340756 | | |  |
| cg14939152Island | | -0.2507246 | | 0.06214557 | | |  |
| cg18068240Island | | 0.05837338 | | 0.22414056 | | |  |
| cg21209684Island | | -0.194978 | | 0.0849479 | | |  |
| cg01967637Island | | 0.14668135 | | -0.036122 | | |  |
| cg22402730Island | | -0.2365491 | | -0.3290018 | | |  |
| cg19135245Island | | -0.0995412 | | 0.39526938 | | |  |
| cg26464411Island | | 0.27988207 | | 0.22879005 | | |  |
| cg07515400Island | | 0.08876877 | | -0.0691418 | | |  |
| cg06968181Island | | -0.01231 | | 0.54912352 | | |  |
| cg18849621Island | | 0.11662806 | | -0.259631 | | |  |
| cg16335926Island | | -0.0279278 | | 0.05804791 | | |  |
| cg10847032Island | | -0.6240987 | | 0.21279284 | | |  |
| cg21702128Island | | 1.15036143 | | -0.4434836 | | |  |
| cg14558428Island | | -0.5263174 | | 0.05235186 | | |  |
| cg24026230S_Shore | | -0.2421351 | | -0.0950428 | | |  |
| cg13648501S_Shore | | 0.04839954 | | 0.07684558 | | |  |
| cg18718518S_Shore | | 0.64672768 | | 0.62032161 | | |  |
| cg13764763S_Shore | | 0.03577352 | | -0.3107141 | | |  |
| cg27345592S_Shore | | -0.0903505 | | 0.11465174 | | |  |
| cg07528216S_Shelf | | -0.4229351 | | 0.1614368 | | |  |
| cg16224829OpenSea | | -0.1804994 | | 0.02081734 | | |  |
| cg01751279OpenSea | | -0.2499042 | | -0.0863047 | | |  |
| cg23430507OpenSea | | 0.18469457 | | -0.0378168 | | |  |
| cg17349736OpenSea | | -0.3826093 | | -0.1024642 | | |  |
| cg14438279OpenSea | | -0.6329859 | | 0.242547 | | |  |
| cg25579735OpenSea | | -0.0966676 | | -0.2799676 | | |  |
| cg08423118OpenSea | | 0.21931333 | | 0.01985263 | | |  |
| cg23776787OpenSea | | -0.0657808 | | 0.06669831 | | |  |
| cg03906910OpenSea | | -0.0307261 | | -0.0366242 | | |  |
| cg08818984OpenSea | | -0.0203066 | | 0.06853196 | | |  |
| cg26720913OpenSea | | -0.1136207 | | -0.0179211 | | |  |
| cg07589972OpenSea | | 0.89737625 | | 0.76923044 | | |  |
| cg12466613OpenSea | | 0.14324952 | | 0.09011001 | | |  |
| cg21979215OpenSea | | -0.0347558 | | 0.00299113 | | |  |
| cg01294526OpenSea | | -0.071142 | | -0.0616804 | | |  |
| cg11022710OpenSea | | -0.2607231 | | -0.0541958 | | |  |
| cg26222722OpenSea | | 0.02440284 | | 0.46955723 | | |  |
| cg10190339OpenSea | | 0.05139366 | | -0.6963958 | | |  |
| cg19828316OpenSea | | 0.01728534 | | -0.107954 | | |  |
| cg06770322OpenSea | | -0.0264473 | | -0.3094267 | | |  |
| cg07637989OpenSea | | -0.1472653 | | 0.13536777 | | |  |
| cg03778647OpenSea | | -0.047395 | | -0.1917222 | | |  |
| cg08320082OpenSea | | 0.07219335 | | -0.0204671 | | |  |
| cg13907255OpenSea | | 0.11386045 | | 0.17453522 | | |  |
| cg05048928OpenSea | | -0.7723566 | | -0.4397035 | | |  |
| cg01391283OpenSea | | 0.38217945 | | 0.38912358 | | |  |
| cg21030557OpenSea | | -0.432312 | | 0.36986793 | | |  |
| cg25553328OpenSea | | 0.27199461 | | 0.36484403 | | |  |
| cg23328217OpenSea | | 0.04675066 | | 0.22041069 | | |  |
| cg19641581OpenSea | | 0.5616814 | | -0.4332995 | | |  |
| cg07197341OpenSea | | 0.14662335 | | 0.04484483 | | |  |
| cg17779063OpenSea | | -0.0433591 | | -0.4794733 | | |  |
| cg17878320OpenSea | | 0.06434329 | | 0.21147088 | | |  |
| cg21334395OpenSea | | 0.08862952 | | -0.013846 | | |  |
| cg13079912OpenSea | | 0.16317126 | | -0.0238397 | | |  |
| cg23484741OpenSea | | 0.10092489 | | -0.19762 | | |  |
